# Disease-Specific Derangement of Circulating Endocannabinoids and *N*-Acylethanolamines in Myeloproliferative Neoplasms

**DOI:** 10.3390/ijms21093399

**Published:** 2020-05-11

**Authors:** Dorian Forte, Flaminia Fanelli, Marco Mezzullo, Martina Barone, Giulia Corradi, Giuseppe Auteri, Daniela Bartoletti, Marina Martello, Emanuela Ottaviani, Carolina Terragna, Antonio Curti, Uberto Pagotto, Francesca Palandri, Michele Cavo, Lucia Catani

**Affiliations:** 1 Institute of Hematology “L. e A. Seràgnoli”, Department of Experimental, Diagnostic and Specialty Medicine (DIMES), School of Medicine, University of Bologna, 40138 Bologna, Italy; martina.barone5@unibo.it (M.B.); giulia.corradi2@unibo.it (G.C.); giuseppe.auteri2@unibo.it (G.A.); daniela.bartoletti3@unibo.it (D.B.); marina.martello2@unibo.it (M.M.); carolina.terragna@unibo.it (C.T.); michele.cavo@unibo.it (M.C.); lucia.catani@unibo.it (L.C.); 2 Unit of Endocrinology and Prevention and Care of Diabetes, Center for Applied Biomedical Research, Department of Medical and Surgical Sciences, University of Bologna, University-Hospital S.Orsola, 40138 Bologna, Italy; marco.mezzullo2@unibo.it (M.M.); uberto.pagotto@unibo.it (U.P.); 3 Institute of Hematology “L. e A. Seràgnoli”, Department of Oncology and Hematology, University-Hospital S.Orsola, 40138 Bologna, Italy; emanuela.ottaviani@aosp.bo.it (E.O.); antonio.curti2@unibo.it (A.C.); francesca.palandri@unibo.it (F.P.)

**Keywords:** endocannabinoids, *N*-acylethanolamines, myeloproliferative neoplasms, myelofibrosis, polycythemia vera, essential thrombocythemia

## Abstract

Growing evidence highlights the endocannabinoid (EC) system involvement in cancer progression. Lipid mediators of this system are secreted by hematopoietic cells, including the ECs 2-arachidonoyl-glycerol (2AG) and arachidonoyl-ethanolamide (AEA), the 2AG metabolite 1AG, and members of N-acylethanolamine (NAE) family—palmitoyl-ethanolamide (PEA) and oleoyl-ethanolamide (OEA). However, the relevance of the EC system in myeloproliferative neoplasms (MPN) was never investigated. We explored the EC plasma profile in 55 MPN patients, including myelofibrosis (MF; *n* = 41), polycythemia vera (PV; *n* = 9), and essential thrombocythemia (ET; *n* = 5) subclasses and in 10 healthy controls (HC). AEA, PEA, OEA, 2AG, and 1AG plasma levels were measured by LC–MS/MS. Overall considered, MPN patients displayed similar EC and NAE levels compared to HC. Nonetheless, AEA levels in MPN were directly associated with the platelet count. MF patients showed higher levels of the sum of 2AG and 1AG compared to ET and PV patients, higher OEA/AEA ratios compared to HC and ET patients, and higher OEA/PEA ratios compared to HC. Furthermore, the sum of 2AG and 1AG positively correlated with *JAK2*^V617F^ variant allele frequency and splenomegaly in MF and was elevated in high-risk PV patients compared to in low-risk PV patients. In conclusion, our work revealed specific alterations of ECs and NAE plasma profile in MPN subclasses and potentially relevant associations with disease severity.

## 1. Introduction

The endocannabinoid (EC) system is composed by the lipid endogenous compounds—*N*-arachidonoyl-ethanolamine (anandamide, AEA) and 2-arachidonoyl-glycerol (2AG), the enzymatic machinery responsible for ligand synthesis and degradation, and the cannabinoid receptors 1 (CB1) and 2 (CB2) [1]. AEA and 2AG are synthesized on demand from membrane phospholipids of many cell types, in the brain and peripheral tissues, as well as in blood cells. Although ECs are supposed to act via paracrine and autocrine communication, their presence in the bloodstream has been quantified and associated with multiple physiopathological conditions [2,3]. CB1 and CB2 are G-protein-coupled receptors; however, CB1 is highly expressed in the central nervous system and in nearly all peripheral tissues, and CB2 is mainly detected in immune cells [4,5]. Both receptors are involved in the regulation of cell proliferation, differentiation, apoptosis, and migration. While AEA acts as a full agonist for both CB1 and CB2, 2AG is a full agonist for CB2 [6].

As a member of the monoacyl-glycerol family, 2AG is an intermediate of diacyl- and triacyl-glycerol metabolic pathways. Due to the poor chemical stability, this compound rapidly and spontaneously isomerizes into 1AG, so that the sum of 2AG and 1AG levels (2+1AG) is often used for evaluating the 2AG biomarker potential in plasma [7,8]. In addition, although long considered inactive, the isomer 1AG was recently shown to have potential agonistic activity supporting 2AG function [9].

AEA belongs to the *N*-acyl-ethanolamide (NAE) family, also including oleoyl-ethanolamine (OEA) and palmitoyl-ethanolamine (PEA) [3]. These signaling lipids share the biosynthetic and degradative machinery as well as non-CB targets, such as the transient receptor potential vanilloid 1 (TRPV1), G-protein-coupled receptors GPR55 and GPR119, and peroxisome proliferator activator receptors (PPAR). NAEs were described to reciprocally influence their activity on target receptors by competing for degrading enzymes, according to an entourage effect mechanism [10,11]. Moreover, although the circulating levels of the three NAEs were found to be highly directly correlated [7,12], imbalances in their relative abundances were associated with their poor metabolic profiles [8,13]. In addition, PPAR-alpha and -gamma targets mediate NAE anti-inflammatory properties [14]. In particular, in contrast to 2AG, which is involved in immune cell recruitment, AEA suppresses pro-inflammatory cytokines production and enhances the release of anti-inflammatory cytokines regulating the immune responses [15,16,17]. Furthermore, PEA was shown to counteract systemic inflammation in mice and humans [14] and to support the increased intestinal permeability associated with inflammation along with OEA [18]. PEA also exhibited immune-modulating properties on different T-cell subsets, thereby representing a new pharmacological player for the treatment of human chronic inflammatory disorders [19].

Interestingly, ECs have been recently found to modulate hematopoiesis, including megakaryocyte maturation, thrombopoiesis, and platelet aggregation, as well as chemokine release and migration of immunocompetent cells [15,20,21,22]. Importantly, blood cells and platelets act as sources of ECs, whereas various hematopoietic cell subsets, particularly B-cells, display high levels of CB2 [23]. In addition, ECs released by platelets are involved in thrombogenic processes [24].

EC system implication in various hematological malignancies has also been investigated [25]. In this regard, high levels of CB2 in hematopoietic precursor cells were shown to exert a role in leukemogenesis [26]. Interestingly, Jorda et al. [27] described that CB2 is expressed in acute myeloid leukemia (AML) blast cells, but not in normal myeloid cells, and that it is associated with migration of bone marrow (BM) precursors mediated by 2AG. Of interest, CB2 revealed oncogenic properties abrogating myeloid differentiation [28]. Recently, interest in the EC system emerged for another hematological malignancy, multiple myeloma (MM). Indeed, it was shown that plasma cells expressed high levels of CB2 and that cannabinoid derivatives selectively induced apoptosis in MM cell lines and primary plasma cells from MM patients [23], similarly to what previously reported for AML [29]. Hence, the EC system in these malignancies might represent a potential target for therapeutic exploitation.

At variance with the mentioned hematological malignancies, to date, no studies investigated the EC system role in myeloproliferative neoplasms (MPN). The MPN include clonal disorders of hemopoietic stem cells such as polycythemia vera (PV), essential thrombocythemia (ET) and primary myelofibrosis (MF) that are driven by mutations in Janus kinase 2 (*JAK2*), myeloproliferative leukemia (*MPL*), or calreticulin (*CALR*) genes [30]; however, none of these could be detected in 2–15% of the patients (triple-negative patients, TN). All MPN are characterized by an increased risk of thromboembolic complications and by the predisposition to evolve into AML. Recently, a selective *JAK1/2* inhibitor, ruxolitinib, was introduced into clinical practice; however, many patients did not respond or did not tolerate this drug [31]. Therefore, more effective therapies are urgently needed.

To our best knowledge, the circulating levels of ECs and related compounds in hematological malignancies have never been reported. Here, for the first time, we investigated the circulating profile defined by levels of the sum of 2AG and 1AG and by the levels and the relative abundances of the NAE AEA, PEA, and OEA in patients affected by MPN, including ET, PV, and MF. In addition, we associated the EC and NAE profile with clinical parameters, mutational status, and disease severity to gain further insight into MPN etiology and to highlight potential disease-related biomarkers.

## 2. Results

### 2.1. Study Cohort

The cohort included 55 patients affected by MPN, recruited at the University Hospital of Bologna, and 10 healthy control (HC) volunteers from the general population. Patients were enrolled at diagnosis or after at least three months from stopping cytotoxic therapy (*n* = 22). MPN patients were subdivided into ET (*n* = 5), PV (*n* = 9), and MF (*n* = 41). Table 1 reports the clinical and laboratory parameters of each class. No differences in sex distribution (*p* = 0.129) were observed, whereas differences were detected in age among classes (*p* < 0.001). MF patients were older (median: 72 years; range: 46–89 years) compared to HC (median: 59 years; range: 31–73 years; *p* = 0.030), ET (median: 52; range: 42–57 years; *p* = 0.005) and PV (median: 57, range: 26–71 years; *p* = 0.002). MPN patients were further stratified into two risk categories: 11 (20%) low-risk (age of <60 years and having no thrombosis history) and 39 (70%) high-risk (age of >60 years and having thrombosis history) patients.

### 2.2. EC and NAE Plasma Profile of MPN Subclasses

**Figure 1:**
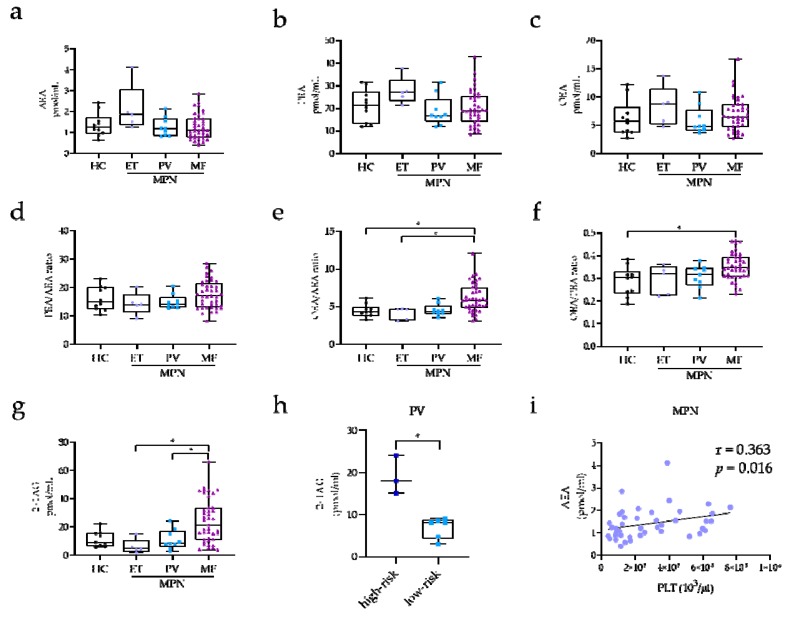
Box-and-whiskers plots for plasma levels of arachidonoyl-ethanolamide (AEA), (**a**) palmitoyl-ethanolamide (PEA); (**b**) oleoyl-ethanolamide (OEA); (**c**) PEA/AEA ratio; (**d**) OEA/AEA ratio; (**e**) OEA/PEA ratio; (**f**) and 1/2-arachidonoyl-glycerol (2+1AG); (**g**) in HC (*n* = 10) and ET (*n* = 5), PV (*n* = 9), and MF (*n* = 41) patients. One-way ANOVA: * *p* ≤ 0.05. (**h**) 2+1AG plasma levels in PV patients at high risk (age of >60 years and/or having thrombosis history; *n* = 3) and low risk (age of <60 years and having no history of thrombosis; *n* = 6). T-test: * *p* ≤ 0.050. (**i**) Pearson’s correlation results between AEA and platelet count (PLT) in MPN patients (*n* = 43).

The levels of AEA, PEA, and OEA and the ratios of PEA/AEA, OEA/AEA, and OEA/PEA, along with the levels of 2+1AG for each MPN subclass and HC are reported in Figure 1. The global analysis of MPN patients showed no significant differences in EC and NAE plasma levels between patients and HC. Additionally, the concentrations of the three NAEs did not significantly vary among MPN classes and HC (AEA: *p* = 0.098; PEA: *p* = 0.203; OEA: *p* = 0.276; Figure 1a–c). However, when NAE ratios were evaluated, significant differences of OEA/AEA and OEA/PEA were found among classes (*p* = 0.001 and *p* = 0.005, respectively). In particular, MF patients exhibited higher OEA/AEA and OEA/PEA ratios as compared to HC (*p* = 0.030 and *p* = 0.010, respectively; Figure 1e–f) and higher OEA/AEA ratios compared to ET (*p* = 0.020). Furthermore, we found that 2+1AG was significantly higher in MF compared to in both ET (*p* = 0.001) and PV patients (*p* = 0.030) (Figure 1g). When PV patients were classified according to risk, high-risk PV patients showed 2-fold increase of 2+1AG levels compared to low-risk PV patients (19.0 ± 2.6 pmol/mL vs. 6.8 ± 0.9 pmol/mL; *p* = 0.030; Figure 1h). Notably, the overall results were not altered, when age was included as a covariate in the analysis.

Circulating levels of 2+1AG and NAEs were not associated with hematological parameters such as white blood cell count, red blood cell count, hemoglobin, platelet count, and hematocrit within MPN classes; however, we observed a direct association of AEA levels with the platelet count when the overall cohort of MPN patients was considered (r = 0.363; *p* = 0.016; Figure 1i).

### 2.3. EC and NAE Plasma Profile According to Risk Classification, Mutational Status, and Clinical Manifestations in MF Patients

**Figure 2:**
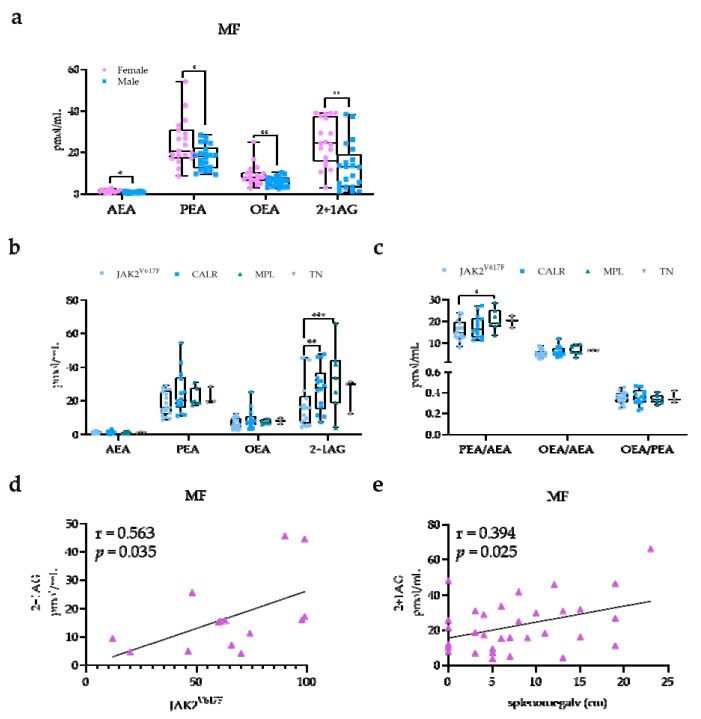
EC and NAE plasma profile according to mutational status and clinical manifestations in MF patients. (**a**) Box-and-whiskers plots for plasma levels in female (*n* = 19) and male (*n* = 21) MF patients. T-test: * *p* ≤ 0.050; ** *p* ≤ 0.010. (**b**) AEA, PEA, OEA and 2+1AG levels; (**c**) PEA/AEA, OEA/AEA, and OEA/PEA ratios according to *JAK2*^V617F^ (*n* = 17), calreticulin (*CALR*; *n* = 14), and myeloproliferative leukemia (*MPL*; *n* = 7) mutational status or triple-negative (TN; *n* = 3). One-way ANOVA: * *p* ≤ 0.050; ** *p* ≤ 0.010; *** *p* ≤ 0.001. Correlation of 2+1AG with (**d**) *JAK2*^V617F^ variant allele frequency (VAF; *n* = 17) and (**e**) splenomegaly (*n* = 41). Pearson’s correlation test.

The large sample size available for the MF class (*n* = 41) allowed us to perform further investigations. Notably, sex differences were observed in MF, with higher NAE and 2+1AG levels in females (*n* = 19) compared with in males (*n* = 21) (AEA, *p* = 0.010; PEA, *p* = 0.022; OEA, *p* = 0.008; 2+1AG, *p* = 0.001; Figure 2a). According to the dynamic international prognostic scoring system (DIPSS) [32], MF patients displayed high risk in five cases (12.1%), intermediate-2 risk in 15 cases (36.6%), intermediate-1 risk in 13 cases (31.7%), and low risk in one case (2.4%). Besides, 15 (36.5%) patients were diagnosed as secondary MF, with five (12.1%) being post-PV MF and 10 (24.3%) being post-ET MF patients. No significant differences were detected in the EC and NAE profile among DIPSS risk categories and between primary and secondary MF.

MF patients were further analyzed according to the mutational spectra as defined by *JAK2*^V617F^ (*n* = 17), *CALR* (*n* = 14), and *MPL* (*n* = 7) mutations and by the absence of these mutations (TN; *n* = 3). We found that *JAK2*^V617F^-mutated MF patients had lower 2+1AG levels compared with patients carrying *CALR* (*p* = 0.006) and *MPL* mutations (*p* < 0.001) (Figure 2b), as well as lower PEA/AEA ratios compared with *MPL*-mutated patients (*p* = 0.003; Figure 2c). Data were still significant when adjusted for age as a covariate. Furthermore, 2+1AG levels were positively correlated with *JAK2*^V617F^ variant allele frequency (VAF) (r = 0.563; *p* = 0.035; Figure 2d), but not with mutant allele burden of *CALR* type 1/2 (*p* = 0.752). Notably, 2+1AG levels were also directly associated with splenomegaly in the overall MF class (*r* = 0.394; *p* = 0.025; Figure 2e).

## 3. Discussion

The EC system is involved in many pathophysiological processes, and its role in cancerogenesis has been postulated [33]. The cancer-associated dysregulation of the EC system might lead to measurable changes in circulating EC levels [34]. Here, we investigated the potential role of the EC system as a disease-specific circulating hallmark of rare MPN.

MPN are known to be characterized by an increased pro-inflammatory status [30,31,35]. Although we did not detect any alteration in circulating levels of AEA, PEA, and OEA in MPN compared to in HC, we reported, for the first time, that AEA concentration was correlated with the platelet count in these patients. Of interest, it has been previously published that AEA in the blood may be one among the factors required for platelet survival [36].

Previous studies demonstrated that circulating concentrations of 2AG are increased in pro-inflammatory states [37,38,39] and are directly correlated with interleukin 6 (IL-6) levels [2,40]. Those findings, on one side, are consistent with our data showing the increased 2+1AG levels associated with the high-risk condition in PV patients. On the other side, the increased 2+1AG levels we described in MF patients could be related to the strong correlation between IL-6 levels and disease severity in MF that we described in our previous study [41]. Additionally, it has been reported that the pathogenesis of MF is linked to the altered megakaryocyte proliferation and differentiation [42,43]. Notably, Gasperi et al. [15] observed that 2AG is a regulator of megakaryocyte/platelet functions. Our findings might suggest a role of this EC in the abnormal megakaryocytopoiesis associated with MF. Furthermore, our data suggested that a potential dysregulation of NAE balance occurs in MF patients in terms of higher OEA/AEA and OEA/PEA ratios. EC and NAE dysregulation have been largely described in obesity and metabolic impairment in humans [12,37]. In previous studies performed in a cohort of healthy subjects from the general population, we reported that 2AG and OEA derangements were associated with insulin resistance and dyslipidemia independently from body mass index (BMI) [8,13]. Another study highlighted how human leukemia cells are able to induce insulin resistance as a mechanism to favor the uncontrolled growth of malignant cells [44]. Whether the dysregulation of the EC and NAE profile we described in MF is related to tumor metabolism and growth deserves further investigations.

Another relevant finding of our study relies on the association of the EC system with the mutational spectrum of MF. For instance, *JAK2*^V617F^-mutated patients displayed lower mean levels of 2+1AG compared to *CALR* and *MPL* mutation carriers. As 2AG is rapidly released in response to pro-inflammatory stimulation of immune cells [45], our results seem to suggest that specific alterations of the immune system depend on the mutational status, as previously reported by our group [46]. On the other hand, 2+1AG was directly correlated with *JAK2*^V617F^ VAF. Most importantly, increasing levels of this EC were associated, in the overall MF cohort, with splenomegaly, a marker of disease severity. These results led us to hypothesize that 2AG levels might be differentially regulated by the three driver-mutated genes in MF and that increasing levels are closely related to disease severity.

Despite the fact that the role of gender in the symptomatology of MPN is still undefined [47,48], Barraco et al. [49] observed that female patients had a specific phenotype with slower disease progression and better prognosis. Here, for the first time, we found higher PEA and 2+1AG plasma levels in MPN female compared to in male patients (data not shown), which was particularly evident in MF subclass, showing a similar trend also for AEA and OEA plasma levels. These features supported the need for gender-specific analysis to better interpret experimental results in MPN.

Altogether, our work highlights the potential involvement of the EC system in the pathophysiology of MPN, further revealing specific associations with features of MPN subclasses. Circulating levels of ECs and related compounds are part of the complex immune-neuro-endocrine system [1,17,34,50], and the present investigation might suggest that this cross-talk is deranged in MPN. Indeed, the depicted alteration in EC and NAE plasma profile might represent a putative biomarker to monitor disease onset and progression in hematological malignancies. Nevertheless, the observations that we reported need to be substantiated in further studies involving larger cohorts of patients and other hematological malignancies, taking into account sex specificities.

In conclusion, our work involving severe and rare hematological malignancies, overall referred to as MPN, revealed for the first time disease-specific alterations of EC and NAE plasma profile, which could help in elucidating the impact of the EC and NAE systems in the pathogenesis, progression, and identification of novel therapeutic strategies.

## 4. Materials and Methods

### 4.1. Study Cohort

All patients and HC gave written informed consent under the approval of the local medical ethical committee of the University Hospital of Bologna (Code 7/2019/Sper/AOUBO of 01/23/2019-Comitato Etico di Area Vasta Emilia Centro), and the study was conducted in accordance with the Declaration of Helsinki. Ten HC from the general population and 55 patients affected by MPN were recruited at the University Hospital of Bologna.

### 4.2. Blood Sampling

Patients and HC gave blood between 8 and 10 a.m,. after overnight fasting. Blood was collected into K2 EDTA-containing tubes (Vacutainer^®^ tubes, Becton Dickinson, Franklin Lakes, NJ, USA) and processed within 1 h from withdrawal. Tubes were centrifuged for 15 min at 3000× *g* to obtain platelet-poor plasma, and the derivative was aliquoted and stored at −80 °C.

### 4.3. Mutation Analysis

*JAK2*^V617F^ allele-burden was assessed in granulocyte DNA with the ipsogen JAK2 MutaQuant Kit (Qiagen, Marseille, France) 505 on the 7900 HT Fast Real-Time PCR System (Applied Biosystem, Monza, Italy). CALR exon 9 sequencing was performed by the next-generation sequencing (NGS) approach with GS Junior (Roche-454 platform; Roche Diagnostics, Monza, Italy); analysis was performed with AVA Software (GRCh38 as referenced). Rare CALR mutations identified by NGS were confirmed by Sanger sequencing. MPL mutations were investigated by the ipsogen MPLW515K/L MutaScreen Kit (Qiagen) and by Sanger sequencing (for MPLS505N and other secondary exon 10 mutations).

### 4.4. EC and NAE Measurements

AEA, PEA, OEA, 1AG, and 2AG plasma levels were measured by a validated in-house assay [7]. Briefly, 0.5 mL of plasma underwent liquid–liquid extraction with 2 mL toluene after the addition of isotopic internal standards. Extracts were injected into the LC–MS/MS platform (HPLC Series200, PerkinElmer, Waltham, Massachusetts; API4000 QTrap, Sciex, Toronto, ON, Canada), separated on a Discovery HS C18 column (7.5 cm × 4.6 mm; particle size: 3 µm), ionized in positive mode by atmospheric pressure chemical ionization and detected by multiple reaction monitoring of both quantitative and confirmation transitions. Baseline separation between 2AG and 1AG isomers was achieved. Functional sensitivities were 0.02 for AEA, 0.20 for PEA and OEA, 0.16 for 2AG, and 0.08 pmol/mL for 1AG.

### 4.5. Statistical Analysis

Mean, SD, frequency, median and range were used as descriptive statistics. PEA/AEA, OEA/AEA, and OEA/PEA molar ratios and the sum of 2AG and 1AG were computed. The normality of variable distribution was analyzed by the Kolmogorov–Smirnov test. All significantly skewed variables showing a positive skewness were transformed according to the equation written as log_10_(x + k), whereas those showing a negative skewness were transformed according to the equation described as the squared root of (x + k). k values resulting in zero skewness after transformations were chosen. Differences in age, sex, and study-specific variables among classes were tested by T-test, ANOVA, and ANCOVA. Specifically, for MF disease, factors other than gender as platelet count (<100 × 10^9^/L), anemia (hemoglobin: <10), peripheral blasts (≥1%), marrow fibrosis grade, large splenomegaly (palpable, ≥10 cm below the left costal margin), and MPN-10 total symptoms score (TSS) (≥20) were considered in univariate analysis. Regression analysis was performed according to the Pearson’s correlation test. The outliers were detected using the Grubbs’s test and excluded from the analyses. *p*-values <0.050 were considered significant. Statistical analyses were performed by Graphpad (Graphpad Software Inc., La Jolla, CA, USA) and by Medcalc version 18.2.1 (MedCalc Software bvba, Ostend, Belgium).

## Figures and Tables

**Table 1:** Clinical and laboratory features of patients within myeloproliferative neoplasms (MPN) subclasses (essential thrombocythemia (ET), polycythemia vera (PV), and myelofibrosis (MF)) and healthy control (HC). Data are expressed as median (range). One-way ANOVA: ET vs. MF: * *p* ≤ 0.050; ** *p* ≤ 0.010; *** *p* ≤ 0.001; PV vs. MF: ^#^ *p* ≤ 0.050; ^##^ *p* ≤ 0.010; ^###^ *p* ≤ 0.001; HC vs. MF: ^+^ *p* ≤ 0.050; ^++^ *p* ≤ 0.010; ^+++^ *p* ≤ 0.001. F: female, M: male, WBC: white blood cell count, PLT: platelet count, Hgb: hemoglobin, RBC: red blood cell count, Hct: hematocrit.

Descriptive Parameters	HC (*n* = 10)	ET (*n* = 5)	PV (*n* = 9)	MF (*n* = 41)
Sex (F/M)	5/5	2/3	3/6	19/22
Age (years)	59 (31–73)	52 (42–57) **	57 (26–71) ^##^	72 (46–89) ^+^
WBC (10^3^/μL)	6.1 (4.3–9.0)	7.5 (7.4–10.3)	8.1 (7.4–14.9)	9.9 (1.6–38.6)
PLT (10^3^/µL)	261 (159–306)	463 (330–656) *	438 (229–762)	121 (38–632)
Hgb (g/dL)	14.1 (12.9–15.5)	14.2 (14–15.4) ***	13.9 (11–16.20) ^##^	9.9 (7.2–15.28) ^+++^
RBC (10^6^/µL)	4.6 (4.1–5.3)	5.59 (4.6–5.65)	5.5 (3.38–7.4) ^#^	3.7(2.4–6.2)
Hct (%)	41.5 (37.6–46.7)	44.7(43–47.98) ***	46.5 (41.8–49.5) ^###^	30.87 (24.16–50.69) ^++^

## Abbreviations

MPN	myeloproliferative neoplasms
MF	myelofibrosis
ET	essential thrombocythemia
PV	polycythemia vera
HC	healthy control
EC	endocannabinoid
NAE	N-acylethanolamide
AEA	N-arachidonoyl-ethanolamine (anandamide)
1AG	1-arachidonoyl-glycerol
2AG	2-arachidonoyl-glycerol
2+1AG	1/2-arachidonoyl-glycerol
PEA	palmitoyl-ethanolamide
OEA	oleoyl-ethanolamide
LC–MS/MS	liquid chromatography–tandem mass spectrometry
CB1/2	cannabinoid receptors ½
WBC	white blood cell
PLT	platelet count
Hct	hematocrit
RBC	red blood cell
Hgb	hemoglobin
TSS	total symptoms score
DIPSS	dynamic international prognostic scoring system
JAK2	janus kinase 2
CALR	calreticulin
MPL	myeloproliferative leukemia protein
TN	triple-negative
VAF	variant allele frequency
BMI	body mass index
